# The complete chloroplast genome sequence of *Castanopsis sclerophylla* (Lindl.) Schott (Fagaceae)

**DOI:** 10.1080/23802359.2020.1790324

**Published:** 2020-07-15

**Authors:** Jing Miao, Yabo Wang, Yaoqin Zhang, Lili Tong, Gengguo Tang, Xiaogang Xu

**Affiliations:** a Co-Innovation Center for Sustainable Forestry in Southern China, College of Biology and the Environment, Key Laboratory of State Forestry and Grassland Administration on Subtropical Forest Biodiversity Conservation, Nanjing Forestry University, Nanjing, PR China; b State Environmental Protection Scientific Observation and Research Station for Ecology and Environment of Wuyi Mountains, Nanjing, PR China; c School of Horticulture & Landscape Architecture, Jinling Institute of Technology, Nanjing, PR China

**Keywords:** *Castanopsis sclerophylla*, phylogenomics, Fagaceae, complete chloroplast genome

## Abstract

*Castanopsis sclerophylla* (Lindl.) Schott is one of the National Class II protected plants, and an important species in subtropical evergreen forests in China. The object of this work was to thoroughly explore the complete chloroplast (cp) genome of *C*. *sclerophylla* using next-generation sequencing. The circular complete cp genome of *C*. *sclerophylla* is 160,519 bp in length, containing a large single-copy (LSC) region of 90,243 bp, and a small single-copy (SSC) region of 18,976 bp. It comprises 131 genes, including 8 rRNA genes, 37 tRNAs genes, and 85 protein-coding genes. The GC content of *C. sclerophylla* cp genome is 36.81%. The phylogenetic analysis suggests that *C*. *sclerophylla* is a sister species to *C*. *fargesii* in Fagaceae.

*Castanopsis sclerophylla* (Lindl.) Schott is the northernmost arbor species of *Castanopsis* Genus. It is a common symbolic species of subtropical evergreen broad-leaved secondary forest between the south of the Yangtze River and the north of Nanling Mountain Area (Chang 1999). *Castanopsis* *sclerophylla* has a lot of economic merits (Chen 2013) like timber, ornamental, food, and other industrial purposes. But, to date, there is not full knowledge on the complete cp genome was measured for *C*. *sclerophylla*. Here, we characterized the complete chloroplast (cp) genome sequence of *C*. *sclerophylla* (GeneBank accession number: MT627605) based on Illumina pair-end sequencing to provide a valuable complete cp genomic resource.

Total genomic DNA was isolated from fresh leaves of an ancient *C*. *sclerophylla* (more than 150 years old) grown in Qixiashan (N 32.1549, E 118.9591), Qixia District, Nanjing, Jiangsu, China. The voucher specimen was deposited at the herbarium of Nanjing Forestry University (accession number NF2020108). The whole-genome sequencing was carried out on Illumina Hiseq platform by Nanjing Genepioneer Biotechnology Inc. (Nanjing, China). The original reading was filtered by CLC Genomics Workbench version 9, and the clean reading was assembled into chloroplast genome with SPAdes (Bankevich et al. 2012). Finally, CpGAVAS (Liu et al. 2012) was used to annotate the gene structure and OGDRAW (Lohse et al. 2013) was used to generate the physical map. Based on the neighbor Joining (NJ), the phylogenetic tree was deduced by MAFFT (Katoh and Standley 2013) and MEGA version 7 (Kumar et al. 2016).

The circular genome of *C*. *sclerophylla* was 160,519 bp in size and contained two inverted repeat (IRa and IRb) regions of 25,650 bp, which were separated by a large single-copy (LSC) region of 90,243 bp, and a small single-copy (SSC) region of 18,976 bp. A total of 131 genes are encoded, including 85 protein-coding genes (79 PCG species), 37 tRNAs gene (30 tRNA species), and 8 rRNA genes (4 rRNA species). Most of the genes occurred in a single copy; however, six protein-coding genes (*ndhB, rpl2, rpl23, rps12, rps7*, *and ycf2*), seven tRNA genes (*trnA-UGC, trnI-CAU, trnI-GAU, trnL-CAA, trnN-GUU, trnR-ACG*, *and trnV-GAC*), and four rRNA genes (*4.5S, 5S, 16S, and 23S*) are totally duplicated. A total of 10 protein-coding genes (*atpF, ndhA, ndhB, petB, petD, rpl16, rpl2, rpoC1, rps12*, *and rps16*) contained one intron while the other two genes (*clpP, ycf3*) had two intron each. The overall GC content of *C*. *sclerophylla* genome is 36.81%, and the corresponding values in LSC, SSC, and IR regions are 34.65%, 30.94%, and 42.79%, respectively.

The phylogenetic analysis was conducted based on 16 Fagaceae cp genomes and 2 taxa (Betulaceae, Myricaceae) as outgroups with sequenced cp genomes. We found that *C*. *sclerophylla* was clustered with other families of Fagaceae with 100% boot-strap values (Figure 1). In addition, *C*. *sclerophylla* was highly supported to be a sister species to *C*. *fargesii* in Fagaceae.

**Figure 1.**
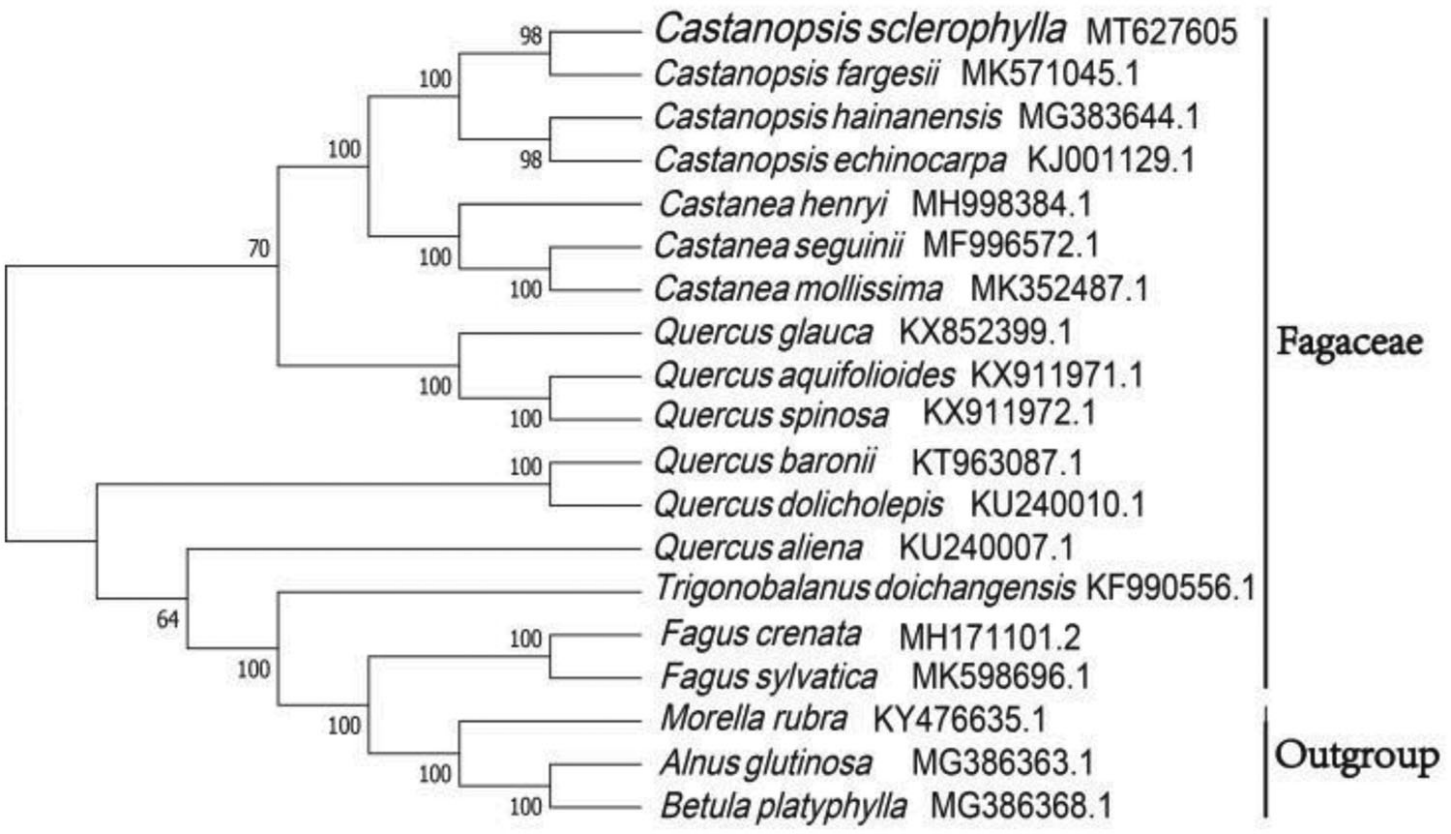
Neighbor-joining tree showing the relationship among *Castanopsis sclerophylla* and representative species within Fagaceae, based on whole chloroplast genome sequences, with two taxa from Fagales as outgroup. The bootstrap support values shown at the branches.

## Data Availability

The data is accessible from: https://pan.baidu.com/s/1f50D7-bBKehSFkuxshUhxw (password：5hsw).
